# The Effect of an Orientation Program Supported by GROW Model–Based Coaching on Nurses’ Turnover Intention, Organizational Commitment, and Performance Levels

**DOI:** 10.1155/jonm/2879299

**Published:** 2026-10-05

**Authors:** Emine Sarıoğlu, Adalet Kutlu

**Affiliations:** ^1^ Department of Nursing Graduate School of Education Manisa Celal Bayar University Manisa Türkiye bayar.edu.tr; ^2^ Department of Nursing Manisa Celal Bayar University Health Sciences College Manisa Türkiye

**Keywords:** coaching, employee performance, organizational commitment, turnover intention

## Abstract

**Aim:**

This study aimed to examine the effect of an orientation program supported by coaching based on the GROW model on nurses’ turnover intention, organizational commitment, and performance levels.

**Background:**

Effective orientation programs support the adaptation of newly recruited nurses to the organization. However, evidence regarding the effects of GROW model–based coaching within orientation programs on nurses’ turnover intention, organizational commitment, and performance is limited.

**Methods:**

This quasiexperimental study with intervention and control groups was conducted between January 2023 and January 2025. In the preliminary phase, interviews were conducted with nurses to determine the topics for pre‐coaching orientation training. Newly recruited nurses were assigned to the intervention group (*n* = 35) and the control group (*n* = 35). Nurses in the intervention group received coaching‐supported orientation training, a face‐to‐face coaching session, and a follow‐up telephone session. Data were collected using a Descriptive Information Form, the Adaptation Performance Scale, Turnover Intention Scale, Organizational Commitment Scale, and Employee Performance Scale. Descriptive statistics, chi‐square test, and Mann–Whitney *U* test were used for data analysis.

**Results:**

The mean Employee Performance Scale score was significantly higher in the intervention group compared to the control group (*p* < 0.05). No significant differences were found between the groups in turnover intention or organizational commitment (*p* > 0.05).

**Conclusions:**

Coaching‐supported orientation training based on the GROW model was found to increase employee performance among nurses. Incorporating coaching‐based educational approaches and appropriate communication styles into nurse orientation programs is recommended.

**Implication for Nursing Management:**

The results indicate that incorporating GROW model–based coaching into nurse orientation programs can enhance the performance of newly recruited nurses. Nursing managers should consider integrating structured coaching practices and effective communication strategies into orientation processes to support nurses’ professional development, facilitate adaptation to the clinical environment, and improve workforce performance.

## 1. Introduction

Organizations should ensure that their employees possess the qualifications necessary to perform their roles effectively. This can be achieved through structured and developmental training practices, particularly orientation programs [1]. Orientation programs play a critical role in helping newly recruited employees understand their responsibilities, become familiar with the organization’s vision, and internalize its culture [2].

Successful adaptation of nurses to a healthcare organization involves more than learning institutional policies and clinical procedures. It also requires the adoption of professional roles, effective communication with team members, development of self‐confidence, and establishment of a sense of organizational belonging. Traditional orientation programs primarily rely on information transfer, preceptorship, and clinical skills training. Although these approaches are effective in developing technical competence, they may be insufficient in supporting individualized development processes such as identifying personal needs, enhancing self‐awareness, providing tailored feedback, and setting personal goals [3]. Consequently, many newly employed nurses continue to experience difficulties adapting to the clinical environment, fear making mistakes, and consider leaving their jobs even after completing orientation programs [4, 5]. Previous studies have shown that effective orientation programs facilitate adaptation to organizational culture, improve confidence and competence, enhance job satisfaction, and reduce stress and turnover among newly employed nurses [2, 6].

To strengthen the effectiveness of orientation programs, developmental approaches such as mentoring and coaching may be incorporated. Coaching is a structured developmental process between a coach and an individual that aims to improve performance and achieve personal and professional goals [7]. It is increasingly recognized as a valuable professional skill capable of enhancing healthcare work environments [8]. Through coaching, individuals become more aware of their strengths and areas requiring development, thereby facilitating workplace adaptation and professional growth. Although coaching has historically received limited attention due to a lack of understanding of its meaning and scope [9], its use in healthcare settings has increased substantially in recent years. Studies suggest that coaching supports professional development, communication skills, teamwork, career planning, change management, and leadership development among nurses [10–12]. Furthermore, Richardson et al. (2023) [13] reported that coaching contributes positively to nurses’ performance, role transition, communication, teamwork, workplace culture, and professional development.

One of the most widely used coaching frameworks is the GROW model, developed by Whitmore (2021) [14]. The model consists of four stages: Goal, Reality, Options, and Will. By encouraging individuals to identify goals, evaluate current circumstances, generate solutions, and commit to action plans, the GROW model promotes self‐reflection, problem‐solving, accountability, and behavioral change. According to Barutçugil (2018) [9], the model supports individuals in generating their own solutions, strengthens responsibility, and facilitates sustainable behavioral change.

From a theoretical perspective, integrating GROW model‐based coaching into orientation programs may enhance nurses′ adaptation to their organizations. Adaptation performance refers to an individual’s ability to effectively adjust to changing work conditions and organizational demands [7, 8]. Nurses who adapt more successfully to their organizations are more likely to identify with organizational goals, demonstrate stronger organizational commitment, and perform their job responsibilities more effectively [15]. Improved adaptation may also reduce uncertainty, workplace stress, and intentions to leave the organization. Therefore, an orientation program supported by GROW model‐based coaching may contribute to stronger organizational commitment and higher performance while reducing turnover intention among newly employed nurses.

Organizational commitment is considered one of the strongest predictors of employees’ intention to remain in or leave an organization. Nurses who develop a strong emotional attachment to their organization, identify with its values, and experience a sense of belonging are less likely to consider leaving their jobs. A comprehensive meta‐analysis reported that all dimensions of organizational commitment were negatively associated with turnover intention, with affective commitment demonstrating the strongest relationship [16]. Similarly, Callado et al. (2023) [17] found a significant negative correlation between organizational commitment and turnover intention among primary healthcare nurses, indicating that nurses with higher levels of commitment were less likely to express intentions to leave their organization. These findings suggest that interventions designed to strengthen organizational commitment may contribute to workforce retention by reducing turnover intention and fostering long‐term organizational engagement.

Although orientation programs have been extensively studied in nursing, the existing literature has primarily focused on traditional orientation, mentorship, preceptorship, and transition‐support models [3, 18]. In contrast, coaching interventions have largely been investigated in the context of leadership development [19], chronic disease management [20], and nursing education [21, 22]. Evidence regarding coaching‐based orientation programs remains limited, particularly studies grounded in a structured coaching framework such as the GROW model. Moreover, few studies have examined the simultaneous effects of coaching‐based orientation on turnover intention, organizational commitment, and employee performance among newly employed nurses.

To the best of our knowledge, no experimental study has evaluated the integration of a GROW model‐based coaching approach into orientation programs while simultaneously examining its effects on nurses’ turnover intention, organizational commitment, and performance levels. The originality of this study lies in integrating a GROW model‐based coaching approach into a nurse orientation program and evaluating its effects on turnover intention, organizational commitment, and performance simultaneously. Therefore, this study aims to address this gap and provide evidence regarding the applicability and effectiveness of GROW‐based coaching within nurse orientation programs.

## 2. Methods

### 2.1. Design and Participants

This study employed a posttest‐only quasiexperimental design with intervention and control groups. The population consisted of all nurses who began working at a Training and Research Hospital during the study period and agreed to participate in the research (*N* = 85). Based on the study data, the required sample size was calculated as 70 participants, assuming an effect size of 0.61 (*d* = 0.61), a significance level of 5% (*α* = 0.05), and a statistical power of 82% (1−*β* = 0.82) [23]. The final sample consisted of 70 nurses, including 35 in the intervention group and 35 in the control group.

Participants were not randomly assigned to the intervention and control groups. This was primarily due to the limited number of newly employed nurses during the study period, which necessitated the inclusion of all eligible nurses. In addition, orientation programs were conducted in accordance with the hospital’s human resources planning, and continuity of healthcare services had to be maintained. Therefore, random allocation was not feasible within the organizational context.

Preliminary interviews with seven nurses were conducted solely for the purpose of needs assessment and the development of the orientation program content. These interviews were not included in the main study dataset and did not constitute a qualitative component of the research.

Before the orientation program, the Participant Information Form and the Adaptation Performance Scale (APS) were administered to assess baseline characteristics and group homogeneity. Adaptation performance was evaluated only for this purpose and was not considered a study outcome. The primary study outcomes, namely turnover intention, organizational commitment, and employee performance, were measured 3 months after completion of the orientation program using the Turnover Intention Scale (TIS), Organizational Commitment Scale (OCS), and Employee Performance Scale (EPS). Accordingly, only posttest measurements were obtained for the study outcomes.

The coaching sessions were delivered by the first author, who had completed a certified coaching training program. Nurses in the intervention group received orientation supported by GROW model‐based coaching in addition to the routine orientation program. Nurses in the control group received only the hospital’s routine orientation program, which represented standard institutional practice.

Inclusion Criteria

• Newly employed nurses at the study hospital
• Participation in the orientation program
• Employment during the study period
• Voluntary participation and provision of written informed consent.

Exclusion Criteria

• Previous employment at the same institution
• Failure to complete the orientation program
• Withdrawal from the study or resignation during the follow‐up period
• Incomplete data collection forms.

### 2.2. Hypotheses and Research Questions

 H1: The orientation program supported by coaching based on the GROW model has an effect on nurses’ turnover intention, organizational commitment, and employee performance.

Research Questions:

1. What has an effect of a GROW model–based coaching‐supported orientation program on nurses’ turnover intention?
2. What has an effect of a GROW model–based coaching‐supported orientation program on nurses’ organizational commitment?
3. What has an effect of a GROW model–based coaching‐supported orientation program on nurses’ employee performance?

### 2.3. Instruments

#### 2.3.1. Information Form

The sociodemographic and occupational characteristics collected included age, gender, education level, marital status, date of employment at the institution, date of entry into the profession, unit of employment, total monthly working hours, and working pattern.

#### 2.3.2. APS

The APS developed by Charbonnier‐Voirin and Roussel [24] and adapted into Turkish by Üstün and Büyükbaş [7] has a reliability coefficient of 0.90. Subscale reliability coefficients were reported as 0.88 for innovation, 0.91 for emergency adaptation, 0.92 for interpersonal adaptability, and 0.85 for training efforts. The scale contains 19 items rated on a 5‐point Likert scale (1 = *Strongly Disagree* to 5 = *Strongly Agree*), with possible scores ranging from 19 to 95. Higher scores indicate higher adaptability performance. In this study, Cronbach’s alpha was 0.95 overall and ranged between 0.77 and 0.90 across subscales. This scale was used to determine baseline homogeneity between groups.

#### 2.3.3. TIS

The TIS developed by Mobley et al. (1978), adapted to Turkish by Turgut, Soran, and Ateş [25], consists of three items rated on a 5‐point Likert scale. The original Cronbach’s alpha was 0.904, while the Turkish adaptation reported a value of 0.93. Scores range from 3 to 15, with higher scores indicating a stronger intention to leave the job. In this study, Cronbach’s alpha was found to be 0.82.

#### 2.3.4. EPS

The EPS comprises 19 items grouped into two subdimensions: employee performance and organizational performance. Since the business performance dimension was not relevant to the aim of this study, only the seven‐item employee performance subscale was utilized. The EPS, which was created by Erdoğan [26], drawing on the foundational work of Rahman and Bullock to assess nurses’ performance. Only the employee performance subscale was used in this study. This subscale contains 7 items rated on a 5‐point Likert scale (1 = Strongly disagree to 5 = Strongly agree), with total scores ranging from 7 to 35. Higher scores indicate better employee performance. The original internal consistency coefficient was 0.87. In the study by Bingöl and Kutlu, the internal consistency coefficient of the EPS was calculated as 0.72 prior to the training and 0.73 following the training, and in this study, Cronbach’s alpha was 0.77 [27].

#### 2.3.5. OCS

Developed by Meyer et al. (1993) and adapted into Turkish by Wasti [16], the scale includes 18 items measuring affective, normative, and continuance commitment. Items 3, 8, 11, and 14 are reverse‐scored. Subscale reliability coefficients in the original adaptation ranged from 0.58 to 0.79. In this study, Cronbach’s alpha was 0.74 overall and ranged from 0.44 to 0.83 across subscales. Scores range from 18 to 90, with higher scores indicating stronger organizational commitment.

### 2.4. Ethical Considerations

Ethical approval was obtained from the Health Sciences Ethics Committee of Manisa Celal Bayar University (Decision date: 14.03.2023; Decision no: 20.478.486/1754). Institutional permission and author permissions for scale use were obtained. All participating nurses signed an informed consent form. Since data collection from the control group lasted 1 year and their institutional orientation was completed during this period, no coaching session or adaptation training was provided to the control group afterward. All procedures adhered to the Helsinki Declaration principles.

Although providing the intervention to the control group after data collection was considered, this was not feasible due to institutional and operational constraints. In addition, the coaching‐supported orientation program was specifically designed to support nurses during their initial transition and adaptation period. Therefore, delayed implementation would not have been consistent with the primary purpose and timing of the intervention. Nurses in the control group received the hospital’s routine orientation program, which represented the standard orientation practice for newly employed nurses.

### 2.5. Data Collection Procedure

As shown in Table 1, the data collection process was conducted in two sequential phases.

**TABLE 1:** Nurses’ opinions and suggestions expressed during coaching sessions regarding orientation training.

Nurses’ opinions and suggestions
“I would like to receive training from a single preceptor within a structured plan. Working with different nurses confuses me.” “My questions should be answered clearly, and I want understanding when I ask questions; otherwise, I avoid asking.” “Because of nurse shortages, I had to start taking patients without sufficient time with my preceptor; I am struggling.”
“I want to be listened to without being judged.” “I want to be asked whether I am adapting well.” “I expect the charge nurse to remain calm, be understanding, and provide constructive feedback; otherwise, my motivation declines.”
“I prefer starting in a less busy unit and later transitioning to a high‐acuity one. I fear making mistakes.” “I came from a small state hospital; working with many residents and physicians has been difficult.” “I have never worked in this unit before and have never encountered a dying patient. This frightens me. I work alone during night shifts and need support in such situations.” “I would like to work with calm and well‐explaining nurses; our opinions should also be considered.” “I have difficulty communicating with staff and feel lonely.” “I need a longer orientation period. I have never worked in such an intense unit and fear making mistakes.”
“Presentations explaining nursing interventions and diseases would be helpful.” “I can research unfamiliar diseases online or purchase books.” “The unit I work in is well‐organized, and educational materials facilitate my orientation.” “I can use note‐taking, journaling, or recording videos to support my learning.” “I can research and learn about diseases (books, AI tools, internet sources, clinical training materials).”

In the first phase, interviews were conducted with seven experienced nurses employed at the study hospital to assess orientation‐related training needs and to develop the content of the coaching‐supported orientation program. These interviews were not part of the main study dataset and did not constitute a qualitative component of the study. The nurses were asked two questions: *“What training topics would you like to receive during orientation?” and “Which instructional methods do you prefer?”* Based on the identified needs and preferences, the content of the coaching‐supported orientation program was table developed.

In the second phase, data were collected from nurses in the intervention and control groups. Before the orientation program commenced, all participants completed the Participant Information Form and the APS to assess baseline characteristics and group homogeneity. Nurses in the control group received the hospital’s routine orientation program, which represented standard institutional practice. Nurses in the intervention group received the same routine orientation program supplemented with a GROW model‐based coaching intervention.

The orientation content identified during the needs assessment was delivered to nurses in the intervention group during their first week of employment. Subsequently, individualized face‐to‐face coaching sessions based on the GROW model were conducted during the second or third week. Each coaching session lasted approximately 45 min. To reinforce the coaching process, a follow‐up telephone coaching session was conducted 1 week after the face‐to‐face session. All coaching sessions were delivered by the first author, who had completed certified coaching training. During the adaptation of the GROW model to the orientation program, methodological support was obtained from an expert trainer who provided coaching certification training.

Three months after completion of the orientation program, the study outcomes were assessed in both groups. At this stage, participants completed the information Form, APS, OCS, EPS, and TIS were administered to all nurses. These measures were used to evaluate the effects of the intervention on turnover intention, organizational commitment, and employee performance.

### 2.6. Coaching Session Structure (GROW Model)

The coaching session was conducted in accordance with the GROW model [17]. The primary aim of the session was to facilitate your orientation to the institution/unit. At the outset, permission was obtained to conduct a 45‐min coaching session. Your current orientation status was evaluated through guiding questions such as: “What does orientation mean to you? Under what circumstances would you consider yourself fully oriented?” and “On a scale of 1–10, where would you position yourself at this point?” Subsequently, strategies to enhance your orientation process were explored, and specific, actionable steps were collaboratively identified. The session concluded with reflective feedback and positive reinforcement to consolidate the established goals and support ongoing professional development.

### 2.7. Data Analysis

Data were analyzed using SPSS 22.0. Descriptive statistics (frequency, percentage, mean, and standard deviation), chi‐square tests, and Mann–Whitney *U* tests were used. Since the data were not normally distributed, regression analysis was not performed. Cronbach’s alpha coefficients were computed for all scales.

## 3. Results

### 3.1. Comparison of Sociodemographic Characteristics Between Groups

No statistically significant differences were found between the intervention and control groups regarding sociodemographic characteristics (*p* > 0.05). In the intervention group, 54.3% of the nurses were under 35 years old, 88.6% were female, 77.1% held a bachelor’s degree, 71.4% worked in rotating shifts, 60% were married, and all nurses (100%) reported willingly choosing the institution. Similarly, in the control group, 57.1% were under 35 years old, 85.7% were female, 77.1% held a bachelor’s degree, 68.6% worked in rotating shifts, 57.1% were married, and all were willing recruits (Table 2).

**TABLE 2:** Comparison of nurses’ sociodemographic characteristics between groups (*n* = 70).

Characteristics	Intervention (*n* = 35)		Control (*n* = 35)		*x* ^2^/*p*
	*n*	%	*n*	%	
Mean of age	36.02 ± 9.09		35.11 ± 9.11		
Age					
< 35 years	19	54.3	20	57.1	0.058/0.810
≥ 35 years	16	45.7	15	42.9	
Gender					
Male	4	11.4	5	14.3	0.128/1.000^#^
Female	31	88.6	30	85.7	
Education					
HS/Assoc	2	5.8	3	8.6	0.291/0.865
BS	27	77.1	27	77.1	
Graduate	6	17.1	5	14.3	
Marital Status					
Married	21	60.0	20	57.1	0.059/0.808
Single	14	40.0	15	42.9	
Working					
Day only	10	28.6	11	31.4	0.068/0.794
Day + Night	25	71.4	24	68.6	

	**Mean ± sd**		**Mean ± sd**		**z/** **p**

Monthly working hours	191.20 ± 22.14		183.08 ± 16.29		−1.532/0.126
Years in profession	11.51 ± 9.44		11.77 ± 10.99		−0.308/0.758

*Note:* *χ* ^2^ = chi‐square test; z = Mann–Whitney *U* test.

^#^Fisher’s exact test.

Three months after starting their positions, the nurses’ turnover intention, organizational commitment, and employee performance levels were compared.

There were no significant differences between the intervention and control groups in terms of TIS scores (*p* > 0.05).

Similarly, OCS total and subscale scores (affective, normative, and continuance commitment) showed no significant differences between groups (*p* > 0.05).

However, EPS scores were significantly higher in the intervention group compared with the control group (*p* < 0.05), indicating that the GROW model–based coaching‐supported orientation program positively contributed to employee performance (Table 3).

**TABLE 3:** Comparison of turnover intention, organizational commitment, and employee performance scores between groups at 3 months (*n* = 70).

Scales		Intervention (*n* = 35)		Control (*n* = 35)		z/*p*
		Mean ± sd	Median (IQR)	Mean ± sd	Median (IQR)	
Turnover Intention Scale		2.25 ± 1.01	2.00 (1.67)	2.40 ± 1.04	2.33 (2.00)	−0.647/0.518
Organizational Commitment Scale (Total)		3.01 ± 0.46	3.05 (0.56)	2.95 ± 0.50	3.05 (0.56)	−0.029/0.977
Organizational Commitment Scale Subdimensions	Affective Commitment	3.15 ± 0.89	3.33 (1.17)	3.02 ± 0.85	3.16 (0.83)	−0.559/0.576
	Normative Commitment	3.01 ± 0.66	3.00 (0.67)	2,93 ± 0,67	2,83 (1.00)	−0.478/0.633
	Continuance Commitment	2.87 ± 0.58	3.00 (1.00)	2.90 ± 0.55	3.00 (0.50)	−0.083/0.934
Employee Performance Scale		4.20 ± 0.49	4.28 (0.71)	3.84 ± 0.66	3.85 (0.57)	**−2.391/0.017** ^∗^

*Note: z*: Mann–Whitney *U* test.

Abbreviation: IQR, interquartile range.

^∗^ *p* < 0.05.

Specifically, the intervention group exhibited higher mean performance scores (4.20 ± 0.49) than the control group (3.84 ± 0.66), demonstrating a statistically meaningful improvement (*p* = 0.017).

## 4. Discussion

### 4.1. Nurses’ Perspectives on the GROW Model–Based Coaching‐Supported Orientation Process

The absence of significant differences between the intervention and control groups in terms of sociodemographic characteristics and baseline APS scores suggests that the groups were comparable at the beginning of the study. During the coaching sessions, nurses shared their experiences, perceptions, and suggestions regarding the orientation process. Participants emphasized that effective communication, a supportive attitude, regular feedback, nonjudgmental listening, and managerial support were critical factors facilitating successful adaptation to the clinical environment.

These findings are consistent with previous studies highlighting the importance of supportive interpersonal relationships during nurses’ transition to practice. Richardson et al. [13] reported that coaching promotes communication, professional development, role transition, and workplace integration among nurses. Similarly, Gardiner and Sheen [28] emphasized that effective communication, frequent feedback, and ongoing support from educators reduce anxiety and facilitate adaptation among newly employed nurses.

Nurses in the present study expressed a preference for receiving orientation from a single clinical educator, indicating that guidance from multiple educators sometimes complicated their adaptation process. Similar findings were reported by Burry et al. [29], who found that newly graduated nurses valued continuity in mentor relationships and experienced difficulties maintaining connection and consistency when mentors changed because of shift schedules or absence. However, Pasila et al. [5] noted that preferences may vary among novice nurses, with some favoring exposure to multiple educators because it broadens learning opportunities.

Participants also described concerns about managing emergency situations independently, particularly during night shifts, and uncertainty regarding clinical decision‐making and communication with physicians. Through the coaching sessions, nurses were encouraged to explore potential solutions, including seeking support from supervisors and experienced colleagues in neighboring units. These findings are consistent with Phillips and Hall [30], who reported that newly graduated nurses often experience anxiety related to independent practice and managing multiple responsibilities during their transition period.

Another notable finding was nurses’ expectation of being understood, listened to without judgment, and provided with constructive feedback by educators and managers. These expectations align with the principles of professional coaching and reflect the core elements of the GROW model, including active listening, self‐reflection, awareness development, and solution‐focused goal setting. Similar observations were reported by Phillips and Hall [30], whose orientation program based on Watson’s Theory of Caring emphasized supportive communication, recognition of individual strengths, appreciation, and goal setting. Likewise, Burry et al. [29] highlighted the importance of mutual understanding and supportive relationships during nurses’ transition to practice.

Some participants suggested that the orientation period should be extended beyond the standard two‐to‐three‐week timeframe. Difficulties were primarily attributed to concerns about completing patient care activities on time and fear of making clinical errors. Previous studies have shown substantial variation in orientation program duration. For example, Pertiwi and Hariyati [31] reported that orientation programs for newly graduated nurses ranged from 6 days to 1 year. Similarly, Allen [32] identified core orientation content, including institutional policies, safety procedures, infection prevention, emergency management, pain management, intravenous therapy practices, patient rights, and transfusion protocols, which are largely consistent with the content delivered in the present study.

Overall, the findings suggest that nurses value individualized support, effective communication, continuity in educational guidance, and opportunities for reflection during their transition into professional practice. These elements are central to GROW model‐based coaching and may contribute to a more supportive and effective orientation experience for newly employed nurse.

### 4.2. Discussion of Turnover Intention, Organizational Commitment, and Employee Performance Scores

In this study, nurses’ turnover intention levels were found to be moderate, which is consistent with previous findings reported by Zaheer et al. [33]. Similarly, Aydoğmuş and Özlük [34] found that 50.8% of nurses reported high turnover intention, while Mohammadi et al. [35] reported that 58.55% of nurses expressed an intention to leave their jobs. In a large‐scale study involving 207,636 nurses, Koehler and Olds [36] found that 21.2% intended to leave their positions within one year.

Although no statistically significant difference was observed between the intervention and control groups regarding turnover intention, the mean turnover intention score in the intervention group was 15% lower than that of the control group. This finding is consistent with Al‐Hello et al. [37], who reported that structured transition interventions, including mentorship and guidance, may reduce turnover intention among newly graduated nurses. In contrast, Çamveren [38] found that turnover intention increased following orientation training. The absence of a significant difference in the present study may be related to the relatively short follow‐up period and the multifactorial nature of turnover intention, which is influenced not only by orientation experiences but also by workload, staffing levels, organizational climate, and career expectations.

In the present study, organizational commitment was found to be moderate, consistent with the findings of Üstün and Büyükbaş [7]. Previous studies have shown that organizational commitment is influenced not only by orientation experiences but also by organizational support, leadership style, teamwork, and workplace culture. In the current study, nurses frequently emphasized the importance of managerial support, understanding, and having sufficient time to adapt. Similarly, Bradley and Moore [19] reported that leadership‐focused coaching improved employee engagement and satisfaction, while Üstün and Büyükbaş [7] identified a positive relationship between nurse collaboration and organizational commitment. More recent evidence also suggests that supportive work environments, peer‐support strategies, and transition programs contribute positively to nurses’ engagement and professional integration [39, 40]. Conversely, Çamveren [38] reported a decline in emotional and normative commitment following orientation training, indicating that orientation alone may not be sufficient to strengthen organizational commitment without ongoing organizational and managerial support. Similarly, Kim et al. [41] reported that although transition programs improved clinical competence and professional development, additional organizational strategies were necessary to enhance commitment.

Employee performance scores were significantly higher in the intervention group than in the control group. This finding supports the effectiveness of the GROW model‐based coaching‐supported orientation program and is consistent with the findings of Bingöl and Kutlu [27], who reported performance levels above the moderate range. Coaching interventions have been associated with improvements in critical thinking, leadership skills, problem‐solving abilities, communication, and professional performance. In nursing, Lee et al. [21] described coaching as a structured learning process that promotes experiential learning through interactive engagement rather than direct instruction. Likewise, Romano et al. [42] demonstrated that coaching programs enhanced problem‐solving, decision‐making, motivation, organizational skills, self‐esteem, and stress management among nursing students.

The positive performance outcomes observed in this study may be explained by the mechanisms inherent in the GROW model. Through individualized goal setting, self‐reflection, exploration of alternatives, and action planning, nurses were encouraged to actively participate in their own development process. This interpretation is supported by Norman [22], who reported that coaching approaches based on the GROW models enhanced confidence, skill development, and readiness for clinical practice.

An additional finding emerging from the coaching sessions was the perceived importance of organizational support, positive communication, fairness, clear role expectations, and supportive leadership during the orientation process. Participants emphasized that empathy, regular feedback, and accessibility of managers and senior staff facilitated adaptation and professional development. These findings are consistent with the meta‐analysis by Richardson et al. [13], which demonstrated that coaching interventions improve communication, teamwork, workplace culture, role transition, and professional performance among nurses.

Overall, the findings suggest that integrating GROW model‐based coaching into orientation programs may support nurses’ adaptation to the workplace by enhancing organizational commitment and employee performance while contributing to lower turnover intention. Although the impact on turnover intention did not reach statistical significance, the observed reduction in mean scores suggests that coaching‐supported orientation may have practical value in supporting nurse retention and workplace integration.

Based on the findings of this study, healthcare organizations may benefit from incorporating structured coaching approaches into nurse orientation programs. Orientation activities should be tailored to individual needs, include opportunities for reflection and goal setting, and extend beyond traditional information‐based training. Nurse managers and clinical educators are encouraged to develop coaching competencies and integrate coaching principles into daily leadership practices. Future studies should evaluate the long‐term effects of coaching‐supported orientation programs in different healthcare settings and examine additional outcomes such as self‐efficacy, clinical decision‐making, job stress, resilience, and patient safety.

The coaching sessions conducted in this study revealed that nurses perceived the orientation process within hospital units in various ways. Some nurses experienced greater difficulty during orientation and required more support than others. It was observed that nurses participated voluntarily in the coaching sessions, and by the end of the process, their awareness had increased, enabling them to independently generate practical solutions. These findings suggest that GROW model–based coaching sessions and coaching‐supported orientation training may be effectively integrated into the orientation process of newly hired nurses and can facilitate their adaptation.

## 5. Limitations

This study has several limitations. As the study was conducted in a single hospital, the findings may have limited generalizability. The quasiexperimental design and lack of randomization may have introduced selection bias. In addition, the relatively small sample size and the 3‐month follow‐up period limited the evaluation of long‐term intervention effects. All outcomes were assessed using self‐report measures. Employee performance was evaluated only through self‐assessment and was not supported by objective performance indicators.

## 6. Conclusion

In this study, no significant differences were found between the intervention and control groups in terms of turnover intention and organizational commitment scores. However, a significant difference emerged in EPS scores, with nurses in the intervention group demonstrating higher performance compared to those in the control group. Overall, the orientation program supported by coaching based on the GROW model did not influence nurses’ turnover intention or organizational commitment but did contribute positively to employee performance. Therefore, the H1 hypothesis was partially supported.

Based on the study findings, it is recommended that healthcare institutions be encouraged to formalize coaching practices within their orientation programs and ensure that nurses have access to coaching support during the early stages of their integration. Orientation content should be tailored to individual needs through coaching‐style conversations and include interactive educational methods that go beyond mandatory training. Nurse managers and clinical educators are advised to strengthen their coaching competencies and integrate this approach into daily leadership practices, fostering more supportive relationships with newly hired nurses. Additionally, coaching schedules should be flexible and individualized, and future research should examine the effects of coaching in different settings and on outcomes such as self‐efficacy, clinical decision‐making, stress, and patient safety.

## Author Contributions

Adalet Kutlu: study conception and design; data evaluation, and interpretation. Emine Sarıoğlu: study design, data collection, manuscript writing, and data evaluation.

## Funding

The authors have nothing to report.

## Conflicts of Interest

The authors declare no conflicts of interest.

## Data Availability

The data that support the findings of this study are available from the corresponding author upon reasonable request.
